# RP11-79H23.3 Inhibits the Proliferation and Metastasis of Non-small-cell Lung Cancer Through Promoting miR-29c

**DOI:** 10.1007/s10528-022-10263-y

**Published:** 2022-08-16

**Authors:** Mulin Liu, Chang Liu, Xi Li, Shijun Li

**Affiliations:** 1 Department of Clinical Laboratory, the First Affiliated Hospital of Dalian Medical University, No. 222 Zhongshan Road, Dalian, 116011 Liaoning Province China; 2 Department of Clinical Laboratory, Weifang People’s Hospital, Weifang, 261000 Shandong Province China

**Keywords:** Non-small-cell lung cancer, RP11-79H23.3, miR-29c, Proliferation, Metastasis

## Abstract

Evidences indicate that long non-coding RNAs (lncRNAs) are closely involved and contributed to tumorigenesis and cancer progression. As a novel lncRNA, RP11-79H23.3 was found to be an anti-oncogene in bladder cancer. However, the essential roles and functions of RP11-79H23.3 in non-small-cell lung cancer (NSCLC) remains to be elucidated. Here, loss of functional assay was applied to gain insights into the functions of RP11-79H23.3 on the proliferation and metastasis capabilities of A549 and H1299 cells. Meantime, Real-time PCR was utilized to measure RP11-79H23.3 and miR-29c expression in NSCLC tissues. Dual-luciferase reporter assay, CCK8, colony formation assay, transwell and Western blot were performed to illustrate the potential molecular basis of RP11-79H23.3 in NSCLC. RP11-79H23.3 downregulation facilitated cell proliferation, migration, and invasion of NSCLC. The result of dual-luciferase reporter assay represented a direct interaction of RP11-79H23.3 with miR-29c, which suppressed miR-29c expression that showed inversely correlation in NSCLC. Moreover, RP11-79H23.3 siRNA facilitated the progression of NSCLC partially via regulating the expression of miR-29c and the activation of Wnt/β-catenin signaling pathway. Our findings highlighted that RP11-79H23.3, served as an anti-oncogene, accelerated NSCLC progression through sequestering miR-29c, providing a promising therapeutic target for NSCLC.

## Introduction

Lung cancer is the leading cause and largest contributor to cancer-related death globally (Ti et al. 2022; Miyasaka et al. 2021). Among the subtypes of lung cancer, non-small-cell lung cancer (NSCLC) is the main type with high morbidity and mortality rate, which accounts for more than 85% of the cases (Yang et al. 2022). It is highly aggressive which leads to distant metastasis easily at the advanced stage. Metastasis is a general complication and critical factor that leading to a remarkable shortened survival rate (Choi et al. 2021; Jiang et al. 2021b; Yu et al. 2021). Owing to cancer metastasis and treatment resistance, the mortality rate of NSCLC is increasing which resulting in a low five-year survival rate, only less than 15% (Wang et al. 2022c). In addition, because of the difficulty in monitoring the metastasis and lacking effective tools for early diagnosis, patients with NSCLC often miss the optimal window for therapy. Consequently, it’s urgent to elucidate the molecular basis which revealing the progression of NSCLC to guide clinical decision-making and discover novel therapeutic target to achieve better prognosis.

Long non-coding RNAs (lncRNAs), with no protein-coding capability, are defined as non-coding RNA transcripts longer than 200nt. To data, there are various functional lncRNAs that have been characterized (Adnane et al. 2022). Mounting evidences have illustrated that lncRNAs have significant functions in a wide variety of diseases (Han and Zhang 2022; Hou et al. 2021; Wang et al. 2020), especially cancer. They were abnormally expressed and exerted huge impact on the aggressive properties through acting as pro-oncogenic or anti-oncogenic roles (Liu et al. 2022; Zhang et al. 2022b; Deshpande et al. 2022; Zhou et al. 2021). For instance, Liao et al. (2022) found that LNMAS suppressed the metastasis of cervical cancer through the interaction with HMGB1 and abrogated the chromatin accessibility of TWIST1 and STC1. Li et al. (2022a) reported that GAL promoted the liver metastasis via stabilizing GLUT1, suggesting that GAL-GLUT1 complex could be a novel treatment target for colorectal cancer liver metastasis. Jiang et al. (2021a) showed that SNHG26 was positively correlated with the proliferation, migration, invasion, EMT and cisplatin resistance in tongue squamous cell carcinoma. Cheng et al. (2022) reported that LHFPL3-AS2 upregulation inhibited NSCLC invasion and metastasis with interacting with SFPQ. In this regard, identifying and discovering NSCLC-related lncRNAs could obtain a better and deeper comprehension of NSCLC progression at molecular level. RP11-79H23.3 is a novel lncRNA with 2994nt located at chromosome 8q21.13. It was found that increased RP11-79H23.3 suppressed the angiogenesis, metastasis and tumorigenesis of bladder cancer through acting like a sponge of miR-107 to elevate PTEN expression (Chi et al. 2018). However, the impact of RP11-79H23.3 on the development and progression of NSCLC is unclear.

Here, functional assays were applied to illustrate the influence of RP11-79H23.3 in mediating the progression of NSCLC, which revealed that RP11-79H23.3 siRNA accelerated the growth and metastasis of NSCLC. Dual-luciferase reporter assay verified the direct binding between RP11-79H23.3 and miR-29c, which was inversely correlated. Moreover, RP11-79H23.3 siRNA stimulated the progression of NSCLC through interacting with miR-29c and activating the Wnt/β-catenin signaling pathway. Thus, a novel axis in NSCLC was found in the current study, where RP11-79H23.3 targeted miR-29c and enhanced NSCLC proliferation and metastasis.

## Materials and Methods

### Cell Culture

A549 and H1299 cells, human NSCLC cell lines, were acquired from the ATCC (Manassas, VA, USA), which were maintained in RPMI-1640 medium (Corning, New York, NY, USA) provided with 10% fetal bovine serum (FBS, Gibco, Invitrogen, NY, USA), 100 μg/ml streptomycin and 100U/ml penicillin. Cells were cultured in a humidified incubator with 5% CO_2_ at 37 °C, and the medium was refreshed regularly.

### Cell Transfection

RP11-79H23.3 siRNA was obtained from RiboBio company (Guangzhou, China), while miR-29c mimics and Anti-miR-29c from GenePharma (Shanghai, China). The synthetic RP11-79H23.3 siRNA (20 nM), miR-29c mimics (50 nM), and Anti-miR-29c (50 nM) were mixed with Lipofectamine 2000 (Invitrogen, Shanghai, China) and added into the cell medium according to the instructions of manufacturer. After transfection for 48 h, both RNA and protein samples were extracted for further analysis. The siRNA, miRNA mimics and inhibitor (Anti-miR-29c) sequences applied in the study were as follows: RP11-79H23.3 siRNA-1: GTAACCCTTTCATGTCATT; RP11-79H23.3 siRNA-2: GTTCTCACATCGCTAACAA; RP11-79H23.3 siRNA-3: CCTATTTCTTACCATCCTT; RP11-79H23.3 siRNA-4: ATGACTTCCCTCTCCTAAGT; RP11-79H23.3 siRNA-5: ATATGTGATTCTCAGACCTC; RP11-79H23.3 siRNA-6: TTGGATCCCTAAGTAACTGA. miR-29c mimics: 5′-UAGCACCAUUUGAAAUCGGUUA-3′, 5′-ACCGAUUUCAAAUGGUGCUAUU-3′; Anti-miR-29c: 5′-UAACCGAUUUCAAAUGGUGCUA-3′.

### Cell Proliferation Assay

A cell density of 1 × 10^3^/well was seeded into 96-well plates. After transfection, cells were incubated for 1–5 days. Subsequently, 10 μl of Cell Counting Kit-8 (CCK8) solution (Beyotime Biotechnology, Beijing, China) and 90 μl RPMI-1640 medium were mixed into the cells, which were continuing to incubate in 37 °C for 2 h. Then, the optical density (OD) at 450 nm was detected, and the data were analyzed.

### Colony Formation Assay

A cell density of 1 × 10^3^ was seeded in six-well plates after transfection and continued to culture at 37 °C for 10–14 days. Then, methanol was utilized to fix the surviving colonies (> 50 cells) after washing with PBS, following with crystal violet (0.1%) staining. Pictures were acquired by using the microscope (Olympus, Tokyo, Japan), and colony numbers were counted.

### Wound Healing Assay

NSCLC cells after transfecting with RP11-79H23.3 siRNA, miR-29c mimics, or Anti-miR-29c were plated into six-well plates. Then, cells were scraped with a sterile pipette tip (200 μl) to make a scratch wound and continued to incubate in FBS-free medium after removing the dead cells and debris. The wound areas pictures were captured with an inverted microscope (Olympus, Tokyo, Japan) at different time intervals of 0 or 24 h and analyzed with Image J software. The representative pictures were shown, as well as the statistical analysis. Each experiment was repeated in triplicate.

### Transwell

For migration assay, the transwell inserts (8 μm, Costar, Cambridge, MA) were not precoated with matrigel, while for invasion assay, the inserts need to precoat with matrigel (1 mg/ml, 1:9, 50 μl, BD Biosciences, San Jose, CA, USA). Cells after transfection were plated into the upper chamber of the insert in 200 μl serum-free RPMI-1640 medium with a cell density of 1 × 10^5^/well, and the lower chamber was fulfilled with 800 μl complete medium. After 24 h, the migrated or invaded cells under the insert membrane were fixed with methanol and stained with crystal violet (0.1%) for 20 min. Cells were recorded and counted in five randomly selected fields, and the representative images were shown.

### Western Blot

Protein samples after transfection were harvested with cell lysis buffer containing PMSF (1 mM, Beyotime Biotechnology, Beijing, China) and cocktail (Roche, IN, USA). The concentration of proteins was determined and separated with 12% SDS-PAGE gel. Proteins in the gels were transferred into the nitrocellulose membrane (Millipore, Billerica, MA), and incubated in 5% skim milk for 2 h. After washing with TBST, primary antibodies were utilized at 4 °C overnight: anti-MMP2 (1:2000, ABclonal, Wuhan, China), anti-MMP9 (1:2000, ABclonal, Wuhan, China), anti-TIMP1 (1:500, ABclonal, Wuhan, China), anti-TIMP2 (1:500, ABclonal, Wuhan, China), and anti-GAPDH (1:5000, ABclonal, Wuhan, China). Next day, the horseradish peroxidase-conjugated second antibody was applied (1:5000, Proteintech, Wuhan, China) for 45 min, and the immunoreactive protein bands were visualized with an enhanced chemiluminescence kit (Thermo Fisher Scientific, IL, USA). The relative expression of proteins was quantified, and the gray value of the protein bands was normalized to GAPDH.

### Real-time PCR

Total RNA from NSCLC tissues and cell lines was extracted with TRIzol reagent (Invitrogen, Shanghai, China), and the cDNA synthesis was performed with the PrimeScript RT reagent Kit (TaKaRa, Dalian, China) after RNA concentration was detected. Real-time PCR was conducted with Applied Biosystem 7500 machine (Foster City, CA) using SYBR Premix Ex Taq™ II PCR kit (TaKaRa, Dalian, China). The 2^−△△CT^ method was applied to analyze the RP11-79H23.3 and miR-29c expression. The primer sequences were listed: RP11-79H23.3: 5′-TGGCCTCAGTTAGGACTGCT-3′ (F), 5′-CTGCTTCCGCTCTCTTTCTC-3′ (R); miR-29c: 5′-CAGACCTGTAGCACCATTTGAA-3′ (F), 5′-TATCCTTGTTCACGACTCCTTCAC-3′ (R); GAPDH: 5′-GAAGGTGAAGGTCGGAGTC-3′ (F), 5′-GAAGATGGTGATGGGATTTC-3′ (R); U6: 5′-CGCTTCGGCAGCACATATAC-3′ (F), 5′-TTCACGAATTTGCGTGTCATC-3′ (R).

### Dual-luciferase Report Assay

The pmirGLO dual-luciferase vector (GenePharma, Shanghai, China) containing RP11-79H23.3 sequence was co-transfected with miR-29c mimics into 293 T cells. Meanwhile, the constructed RP11-79H23.3-WT/MUT vectors were co-transfected with miR-29c mimics or miR-29c negative control. After transfection for 48 h, the cell lysates were subjected for the analysis of relative luciferase activity on the dual-luciferase reporter assay system (Promega, Mannheim, Germany).

### Clinical Samples

A total of 31-paired NSCLC tissues (surgical specimens) were acquired from the First Affiliated Hospital of Dalian Medical University from February 2019 to December 2021, who did not receive any radiotherapy or chemotherapy before. The research was approved by the Ethics Committee of the First Affiliated Hospital of Dalian Medical University, and the written informed consent was got from every subject.

### Statistical Analysis

All data in the study got from three independent repeated experiments were represented as the means ± S.E.M. Differences between two groups were analyzed with Student’s *t* test, and the statistical analysis was conducted with SPSS 23.0 (IBM Corp, Armonk, NY, USA) and GraphPad Prism 9 software (GraphPad Software, inc., San Diego, CA, USA). A *P* value < 0.05 was considered as an indicative of significant.

## Results

### Downregulation of RP11-79H23.3 Promoted Cell Proliferation, Migration, and Invasion of NSCLC

**Fig. 1:**
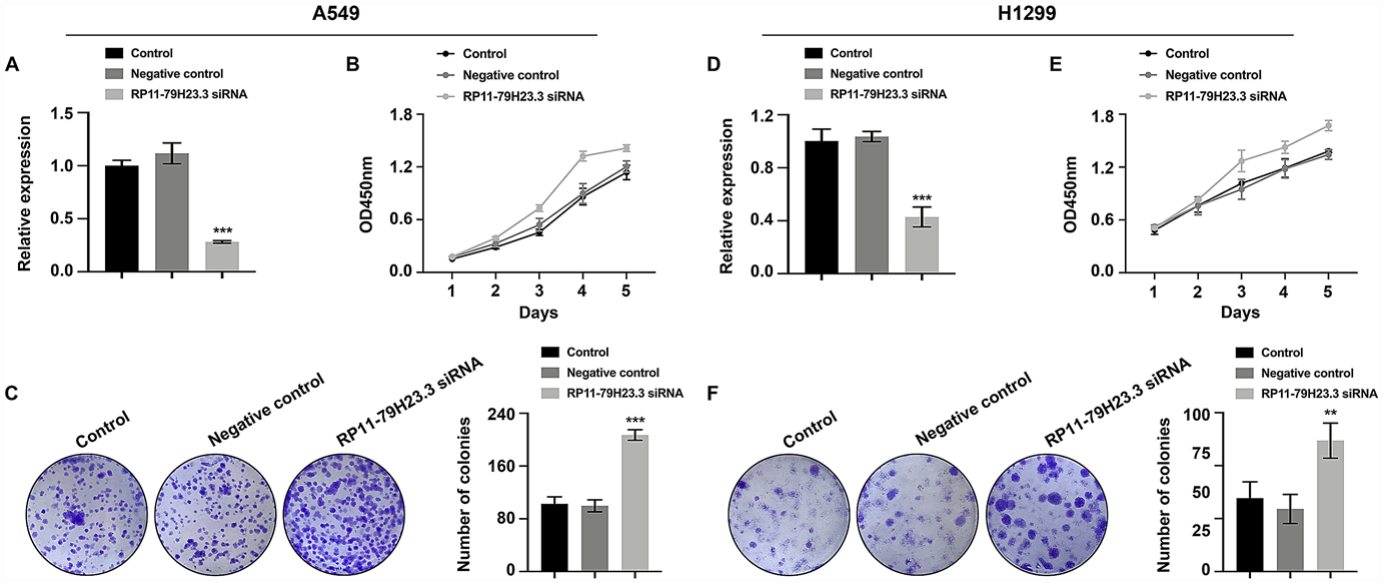
Downregulation of RP11-79H23.3 promoted cell proliferation of NSCLC. Cells were transfected with RP11-79H23.3 siRNA, and the proliferation ability of NSCLC cells was analyzed. **A** and **D** The relative expression of RP11-79H23.3 was detected by Real-time PCR in A549 (**A**) and H1299 (**D**) cells. **B** and **E** CCK8 assay of cell proliferation ability in both A549 (**B**) and H1299 (**E**) cells. **C** and **F** Colony formation assay of cell proliferation rate in A549 (**C**) and H1299 (**F**) cells. **P < 0.01, ***P < 0.001

**Fig. 2:**
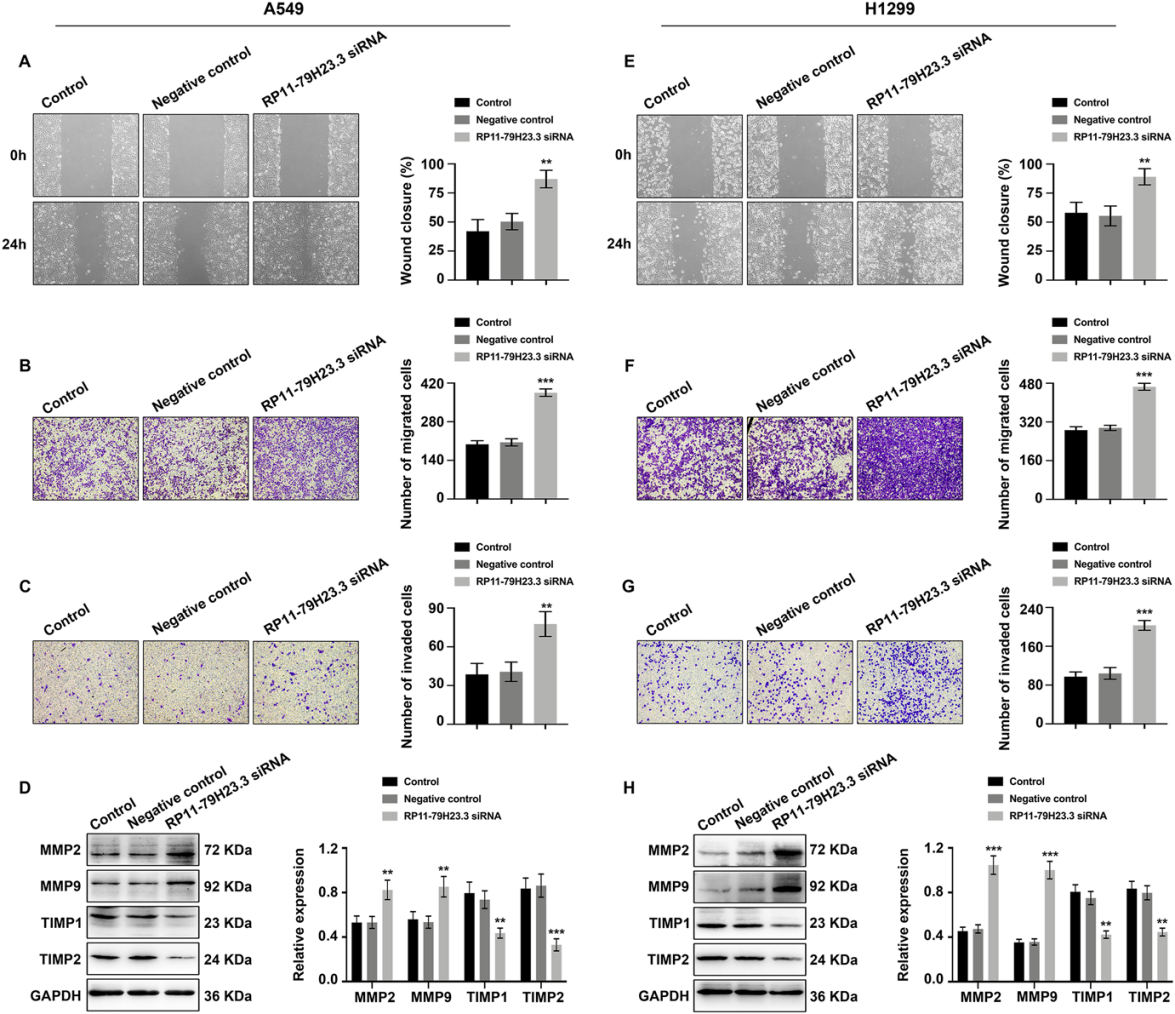
Downregulation of RP11-79H23.3 promoted cell migration and invasion of NSCLC. Cells were transfected with RP11-79H23.3 siRNA, and the migration and invasion capabilities were analyzed. **A** and **E** Wound healing assay of cell migration capability in A549 (**A**) and H1299 (**E**) cells. **B** and **F** Transwell migration assay of cell migration ability in A549 (**B**) and H1299 (**F**) cells. **C** and **G** Transwell invasion assay of cell invasion capability both in A549 (**C**) and H1299 (**G**) cells. **D** and **H** Western blot of tumor metastasis-related proteins (MMP2/9, TIMP1/2) in A549 (**D**) and H1299 (**H**) cells. The statistical analysis was shown. **P < 0.01, ***P < 0.001

To uncover the biological functions of RP11-79H23.3 in NSCLC, RP11-79H23.3 siRNA was used for transfection. Satisfactory transfection efficiency was acquired after 48 h post-transfection (Fig. 1A and D). Compared to negative control group, RP11-79H23.3 siRNA resulted in an evident increase in cell proliferative capability of NSCLC cells which was monitored by CCK8 (Fig. 1B and E) and colony formation assay (Fig. 1C and F). Furthermore, wound healing assay (Fig. 2A and E) and transwell migration assay (Fig. 2B and F) reflected a considerable facilitation in cell migration in RP11-79H23.3 siRNA group. Transwell invasion assay (Fig. 2C and G) and the expression alterations of cancer metastasis-associated proteins (MMP2/9, TIMP1/2) (Fig. 2D and H) showed an accelerative effect of RP11-79H23.3 siRNA on the metastasis capability of NSCLC cells. Consequently, these findings supported that RP11-79H23.3 downregulation exerted promotive effects on cell proliferation and metastasis capabilities of NSCLC cells.

### RP11-79H23.3 Suppressed miR-29c Expression

**Fig. 3:**
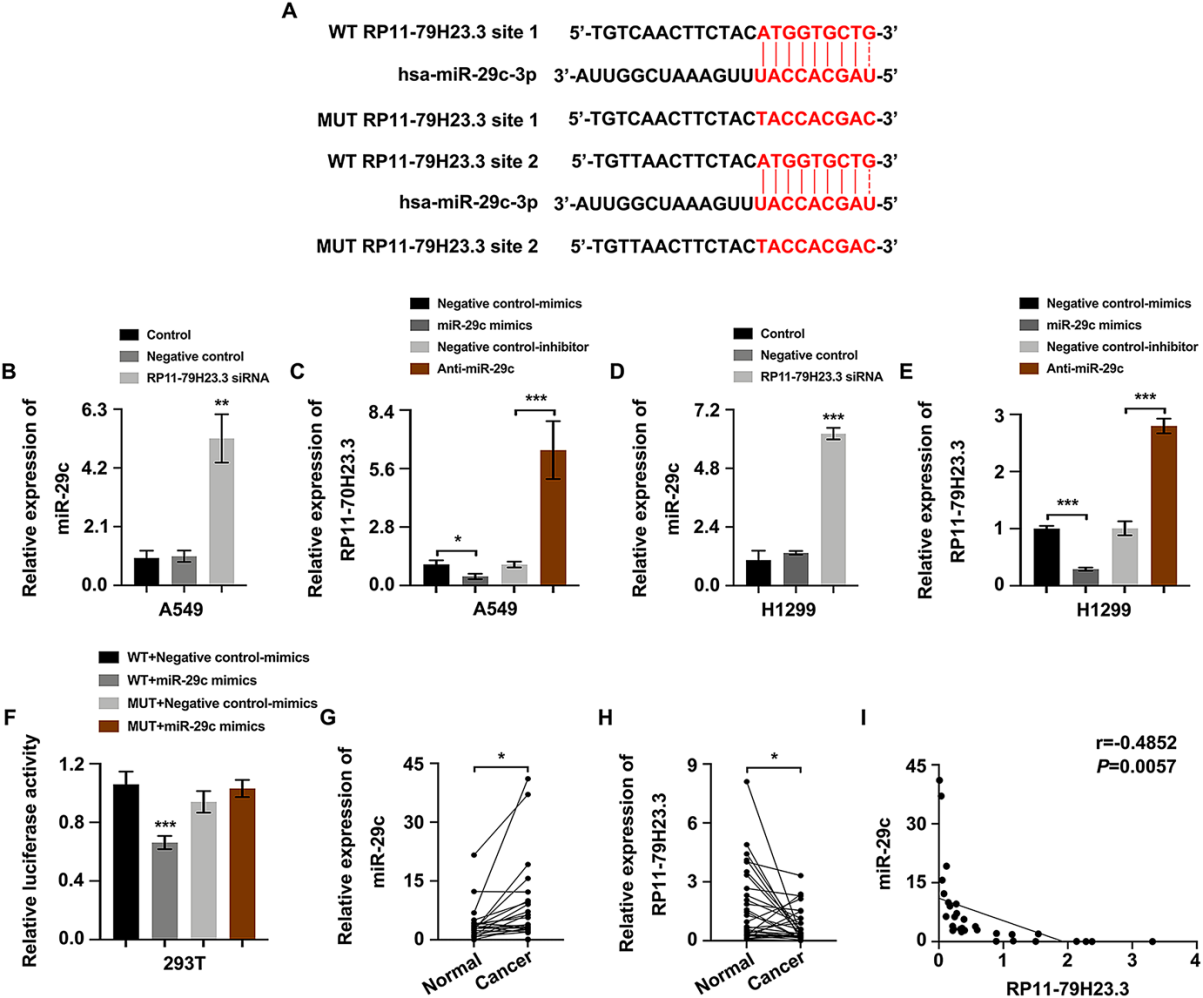
miR-29c was a novel target of RP11-79H23.3. **A** The binding sequence of RP11-79H23.3 and miR-29c. **B** and **D** Real-time PCR of miR-29c expression after RP11-79H23.3 siRNA transfection in A549 (**B**) and H1299 (**D**) cells. **C** and **E** Real-time PCR of RP11-79H23.3 expression after miR-29c mimics and Anti-miR-29c transfection in both A549 (**C**) and H1299 (**E**) cells. **F** Dual-luciferase reporter assay detected the connection between RP11-79H23.3 and miR-29c. **G** and **H** The expression of miR-29c (**G**) and RP11-79H23.3 (**H**) in NSCLC tissues and adjacent non-tumor tissues detected with Real-time PCR. **I** The correlation between RP11-79H23.3 and miR-29c in NSCLC analyzed with Spearman’s correlation analysis. *P < 0.05, **P < 0.01, ***P < 0.001

To explore the functional mechanism of RP11-79H23.3 in NSCLC, the specific target miRNA of RP11-79H23.3 was searched, which turned out to be miR-29c (Fig. 3A). As expected, miR-29c expression was elevated in RP11-79H23.3 siRNA group (Fig. 3B and D), whereas RP11-79H23.3 expression was decreased in miR-29c mimics group or increased in Anti-miR-29c group (Fig. 3C and E). It demonstrated that miR-29c could be modulated by RP11-79H23.3. Moreover, to verify the connection between RP11-79H23.3 and miR-29c, dual-luciferase reporter assay was applied which pointed put that enforced expression of miR-29c could attenuate the luciferase activity of RP11-79H23.3-WT reporter rather than RP11-79H23.3-MUT reporter, implying that RP11-79H23.3 was closely connected with miR-29c which showed directly binding (Fig. 3F). We further determined the expression of miR-29c and RP11-79H23.3 in NSCLC tissues and adjacent non-tumor tissues through Real-time PCR. As shown, miR-29c manifested a higher expression in NSCLC tissues (Fig. 3G), while RP11-79H23.3 represented a lower expression (Fig. 3H), which showed an inversely correlation in NSCLC (Fig. 3I). Taken together, miR-29c was a direct downstream target of RP11-79H23.3.

### miR-29c Promoted Cell Proliferation, Migration, and Invasion of NSCLC

**Fig. 4:**
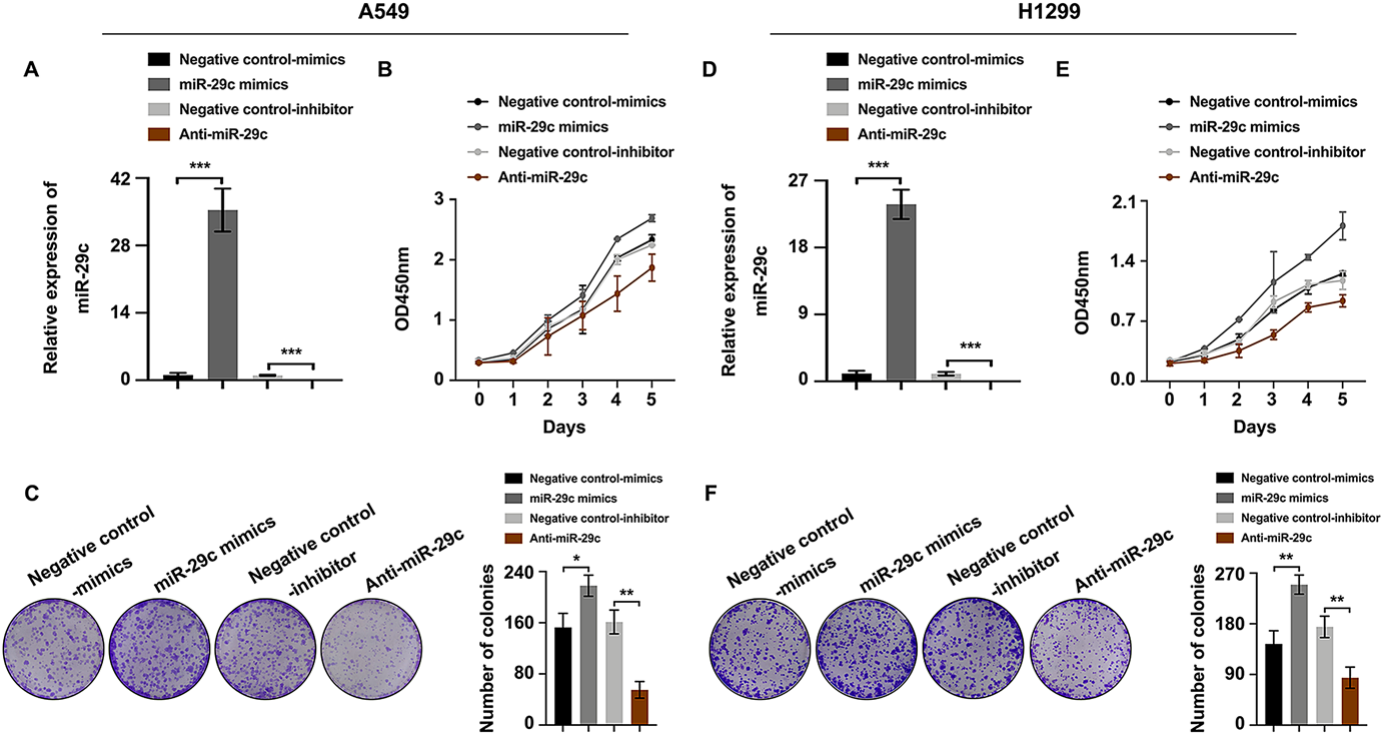
miR-29c promoted cell proliferation of NSCLC. NSCLC cells were transfected with miR-29c mimics and Anti-miR-29c, and the alterations of cell proliferation were detected. **A** and **D** Real-time PCR of miR-29c expression in A549 (**A**) and H1299 (**D**) cells. **B** and **E** CCK8 assay of cell proliferation rate in both A549 (**B**) and H1299 (**E**) cells. **C** and **F** Colony formation assay of the proliferation ability in A549 (**C**) and H1299 (**F**) cells. *P < 0.05, **P < 0.01, ***P < 0.001

**Fig. 5:**
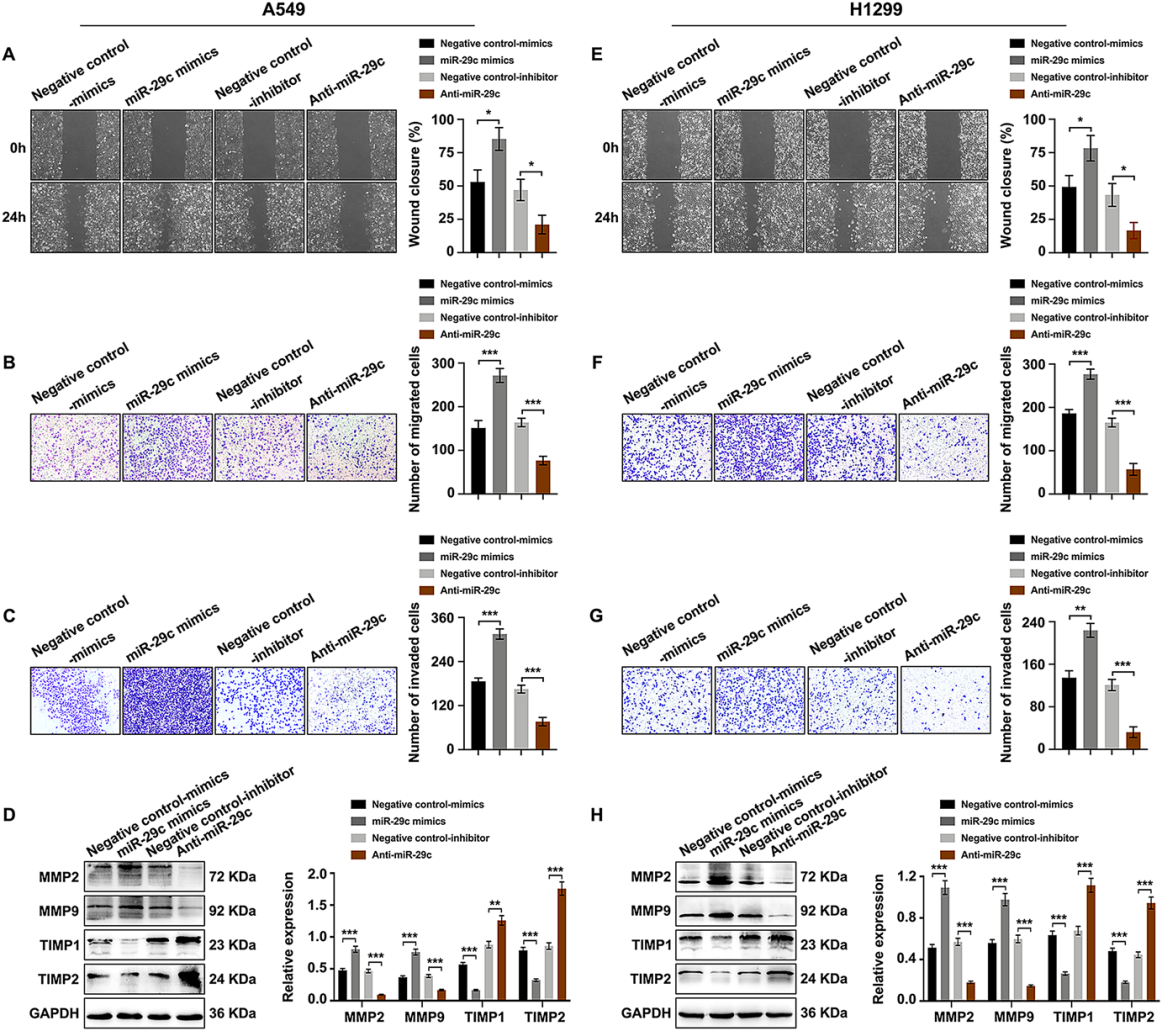
miR-29c promoted cell migration and invasion of NSCLC. NSCLC cells were transfected with miR-29c mimics and Anti-miR-29c, and the migration and invasion abilities were analyzed. **A** and **E** Wound healing assay of cell migration ability in A549 (**A**) and H1299 (**E**) cells. **B** and **F** Transwell migration assay of cell migration capability in A549 (**B**) and H1299 (**F**) cells. **C** and **G** Transwell invasion assay of cell invasion ability in A549 (**C**) and H1299 (**G**) cells. **D** and **H** Western blot analysis of cancer metastasis-associated proteins (MMP2/9, TIMP1/2) in A549 (**D**) and H1299 (**H**) cells. *P < 0.05, **P < 0.01, ***P < 0.001

To illustrate the role of miR-29c in NSCLC development, miR-29c mimics and Anti-miR-29c were transfected into A549 and H1299 cells. Satisfactory transfection efficiency was got after 48 h post-transfection (Fig. 4A and D). When compared to the negative control group, miR-29c mimics lead to an increase while Anti-miR-29c resulted in a decrease in cell proliferation capability of NSCLC cells as detected by CCK8 assay (Fig. 4B and E). These findings were further verified by colony formation assay, revealing that miR-29c promoted cell proliferation whereas Anti-miR-29c inhibited cell proliferation in NSCLC (Fig. 4C and F). In addition, wound healing assay (Fig. 5A and E) and transwell migration assay (Fig. 5B and F) represented a promotive effect in cell migration in miR-29c mimics group whereas the adverse effect was observed in Anti-miR-29c group. Transwell invasion assay (Fig. 5C and G) and the expression changes of cancer metastasis-associated proteins (MMP2/9, TIMP1/2) (Fig. 5D and H) suggested an enforced impact on cell invasion ability in miR-29c mimics group whereas an impaired effect in Anti-miR-29c group. These evidences illustrated that miR-29c promoted NSCLC progression.

### RP11-79H23.3/miR-29c Boosted the Proliferation and Metastasis of NSCLC Through Wnt/β-catenin Signaling Pathway

**Fig. 6:**
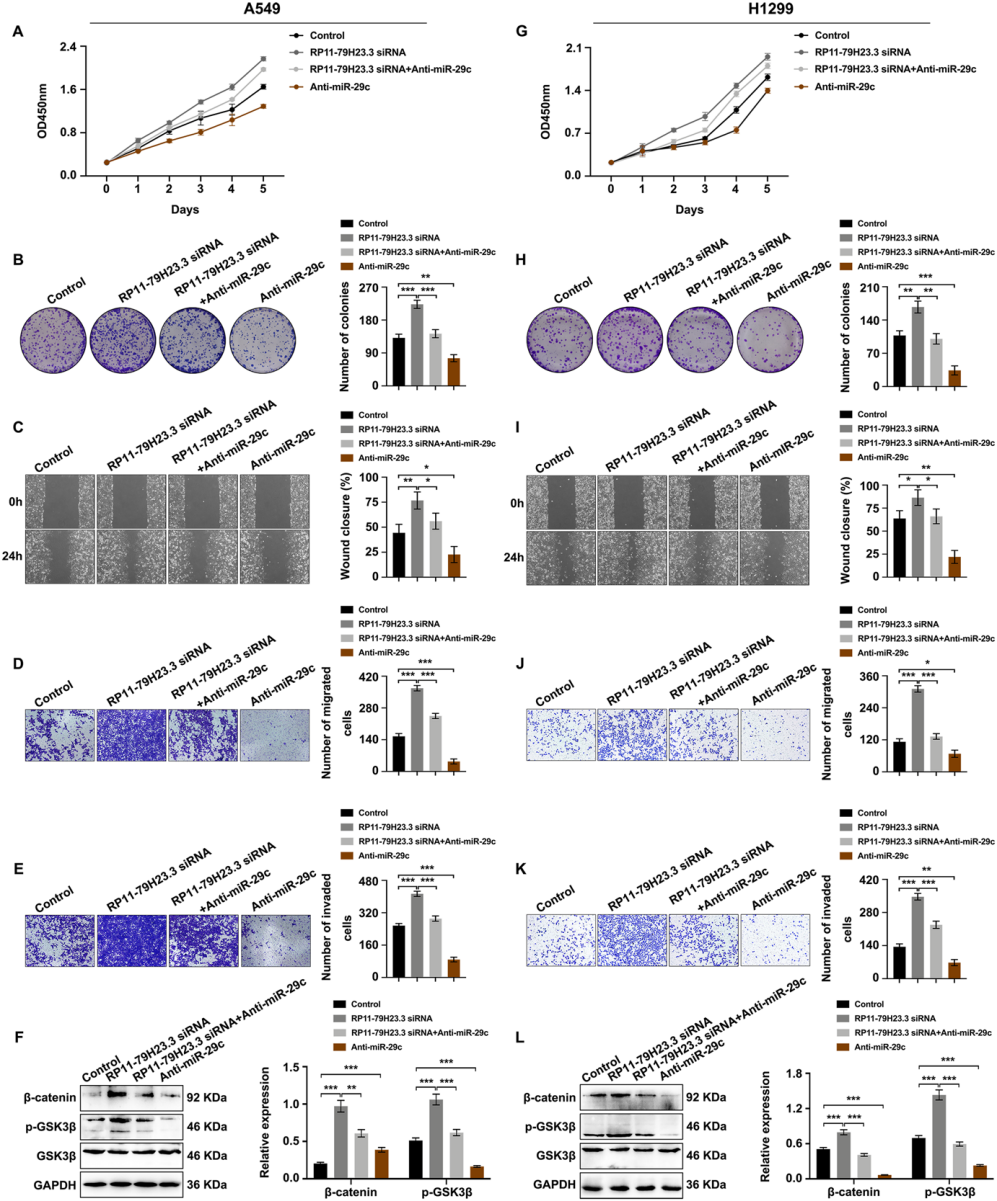
RP11-79H23.3/miR-29c promoted cell proliferation and metastasis of NSCLC through Wnt/β-catenin signaling pathway. Cells were transfected with RP11-79H23.3 siRNA, combined with Anti-miR-29c or Anti-miR-29c transfection alone, and the alterations of proliferation, migration, or invasion abilities, as well as the activation of Wnt/β-catenin signaling pathway were determined. **A** and **G** CCK8 assay of the alterations of cell proliferation rate in A549 (**A**) and H1299 (**G**) cells. **B** and **H** Colony formation assay of cell proliferation in A549 (**B**) and H1299 (**H**) cells. **C** and **I** Wound healing assay of cell migration capability in A549 (**C**) and H1299 (**I**) cells. **D** and **J** Transwell migration assay of cell migration ability in A549 (**D**) and H1299 (**J**) cells. **E** and **K** Transwell invasion assay of cell invasion capability in A549 (**E**) and H1299 (**K**) cells. **F** and **L** Western blot analysis of the activation of Wnt/β-catenin signaling pathway in A549 (**F**) and H1299 (**L**) cells. *P < 0.05, **P < 0.01, ***P < 0.001

To investigate the role of miR-29c in RP11-79H23.3-mediated NSCLC proliferation and metastasis, rescue assays were carried out. As shown, the proliferation ability increased by RP11-79H23.3 downregulation was rescued after miR-29c inhibition (Fig. 6A and G). Colony formation assay validated that promoted cell proliferation induced by RP11-79H23.3 downregulation could be compensated by miR-29c decreasion (Fig. 6B and H). Moreover, reduced expression of miR-29c could restore the accelerated cell migration (Fig. 6C and I, Fig. 6D and J) and invasion (Fig. 6E and K) in RP11-79H23.3 siRNA group. To move on, the activation of Wnt/β-catenin signaling pathway was determined. It was shown that RP11-79H23.3 downregulation activated the Wnt/β-catenin signaling pathway with elevated expression of β-catenin and p-GSK3β, which could be restored after co-transfection with Anti-miR-29c. Anti-miR-29c suppressed the activation of Wnt/β-catenin signaling pathway (Fig. 6F and L). To sum, RP11-79H23.3/miR-29c promoted NSCLC progression through activating the Wnt/β-catenin signaling pathway.

## Discussion

The regulation of lncRNAs in cancer states has several mechanisms, among which ceRNA was the most common one (Chen et al. 2022; Wang et al. 2022a). lncRNA can serve as ceRNA to suppress biological functions through post-transcriptional regulation. Wang et al. (2022b) found that downregulation of OIP5-AS1 promoted the expression of miR-92a to repress cell proliferation and metastasis of ovarian cancer via regulating ITGA6. Zhang et al. (2022a) reported that RNASEH1-AS1 exacerbated its oncogenicity of NSCLC through regulating miR-516a-5p/FOXK1 axis and facilitating Wnt/β-catenin signaling pathway activation. Li et al. (2022b) found that downregulation of LINC01315 modulated the properties of cancer stem cells and EMT of colorectal cancer through miR-484/DLK1 axis. Furthermore, Chi et al. (2018) reported that RP11-79H23.3 could promote bladder cancer progression through sponging miR-107. However, the ceRNA mechanism of RP11-79H23.3 deregulation in NSCLC remains to be illustrated. To gain mechanistic detail, we identified miR-29c as a potential candidate miRNA to RP11-79H23.3 via bioinformatics analysis. We observed that downregulation of RP11-79H23.3 upregulated miR-29c expression in NSCLC cells, whereas upregulation of miR-29c decreased RP11-79H23.3 expression. So we hypothesized that it played functional roles in a miR-29c dependent manner in NSCLC. Dual-luciferase reporter assay was carried out, which confirmed the direct binding between RP11-79H23.3 and miR-29c. Thus, we concluded that RP11-79H23.3/miR-29c axis might exert its roles through ceRNA mechanism in NSCLC.

Evidences have demonstrated that miR-29c was involved in cancer progression (Deshpande et al. 2022; Nai et al. 2022; Li et al. 2021; Hozaka et al. 2021), including NSCLC. For instance, Liu J et al. found that upregulation of miR-29c-3p suppressed CDCA4 expression and decreased cell proliferation, migration, invasion, apoptosis, and EMT of melanoma, which hindering melanoma progression (Liu et al. 2021). Deng M et al. reported that miR-29c enhanced the anti-tumor efficacy of NK cells through regulating B7-H3 directly in ovarian cancer (Deng et al. 2021). Zou et al. (2021) found that miR-29c-3p inhibited EMT process by targeting SPARC and suppressed the metastasis capability of cervical cancer. Sun et al. (2018) reported that miR-29c strengthened the sensitivity of NSCLC cells to cisplatin by activating PI3K/Akt signaling pathway. In this study, miR-29c was shown to act as one downstream target of RP11-79H23.3, accelerating the growth and metastasis of NSCLC cells. Moreover, rescue assays disclosed that the promoted NSCLC cell proliferation and metastasis caused by RP11-79H23.3 siRNA was partially recovered by Anti-miR-29c. Our data revealed that RP11-79H23.3 lead to the growth and metastasis of NSCLC through targeting miR-29c, and RP11-79H23.3/miR-29c axis might be a potential treatment target for NSCLC.

Overall, these findings have expanded our comprehension of RP11-79H23.3 functions as an anti-oncogene in NSCLC. Intriguingly, we identified that dysfunction of RP11-79H23.3/miR-29c axis was closely associated with abnormal cell proliferation and metastasis of NSCLC. A deeper and detailed understanding of the roles of RP11-79H23.3/miR-29c axis might suggest a promising potential therapeutic target for NSCLC.

## Data Availability

The data generated during analyzing in the current study are available from the corresponding author on reasonable request.
